# High tumour islet macrophage infiltration correlates with improved patient survival but not with EGFR mutations, gene copy number or protein expression in resected non-small cell lung cancer

**DOI:** 10.1038/sj.bjc.6604256

**Published:** 2008-02-19

**Authors:** D-W Kim, H S Min, K-H Lee, Y J Kim, D-Y Oh, Y K Jeon, S-H Lee, S-A Im, D H Chung, Y T Kim, T-Y Kim, Y-J Bang, S W Sung, J H Kim, D S Heo

**Affiliations:** 1 Department of Internal Medicine, Seoul National University Hospital, Seoul, Korea; 2 Department of Pathology, Seoul National University Hospital, Seoul, Korea; 3 Department of Thoracic and Cardiovascular Surgery, Seoul National University Hospital, Seoul, Korea; 4 Cancer Research Institute, Seoul National University College of Medicine, Seoul, Korea

**Keywords:** macrophage, non-small cell lung cancer, epidermal growth factor receptor, survival analysis

## Abstract

The purpose of this study was to investigate the prognostic value of tumour-associated macrophages with a focus on micro-anatomical localisation and determine whether molecular changes of the epidermal growth factor receptor (EGFR) are related to macrophage infiltration in resected non-small cell lung cancer (NSCLC). One hundred and forty-four patients were included in this study. Immunohistochemistry was used to identify CD68+ macrophages in the tumour islet and surrounding stroma. Epidermal growth factor receptor mutations were studied by direct sequencing. The EGFR gene copy number and protein expression were analysed by fluorescence *in situ* hybridisation and immunohistochemistry. Patients with a high tumour islet macrophage density survived longer than did the patient with a low tumour islet macrophage density (5-year overall survival rate was 63.9 *vs* 38.9%, *P*=0.0002). A multivariate Cox proportional hazard analysis revealed that the tumour islet macrophage count was an independent prognostic factor for survival (hazard ratio 0.471, 95% confidence interval 0.300–0.740). However, EGFR mutations, gene copy number, and protein expression were not related to the macrophage infiltration. In conclusion, tumour islet macrophage infiltration was identified as a strong favourable independent prognostic marker for survival but not correlated with the molecular changes of the EGFR in patients with resected NSCLC.

It is well known that macrophages infiltrate into tumour tissue (Svennevig and Svaar, 1979). Generally, macrophages were thought to have a tumour cell killing function through secretion of cytotoxic molecules such as tumour necrosis factor-*α* (Bonta and Ben-Efraim, 1993) and nitrogen oxide (Cui *et al*, 1994). However, several experimental findings have supported a role for macrophages in enhancing tumour progression through production of growth and angiogenic factors including the epidermal growth factor, transforming growth factor-*β*, and vascular endothelial growth factor (Xiong *et al*, 1998; Leek and Harris, 2002).

The studies with clinical samples to date also have shown contradictory results. Some studies have shown that macrophage infiltration was associated with a poor prognosis in breast (Leek *et al*, 1996), prostate (Lissbrant *et al*, 2000), and bladder cancer (Hanada *et al*, 2000). However, other studies have shown that macrophage infiltration was associated with good prognosis in prostate (Shimura *et al*, 2000) and colon cancer (Khorana *et al*, 2003).

For the prediction of prognosis in non-small cell lung cancer (NSCLC) patients, the results have also been contradictory. Two studies showed that macrophage infiltration was associated with a poor prognosis (Takanami *et al*, 1999; Chen *et al*, 2003), whereas two other studies showed that macrophage infiltration was not related with prognosis (Johnson *et al*, 2000; Toomey *et al*, 2003). Recently, Welsh *et al* (2005) reported the result of an immunohistochemical study with a focus on the micro-anatomical localisation of macrophages. They showed that the presence of tumour islet macrophages was related with a good prognosis, whereas stromal macrophage infiltration was associated with a poor prognosis in patients with NSCLC. These findings are consistent with the suggested dual function of macrophages identified in prior experimental studies. In addition, clinical studies on gastric, and colon cancer, which analysed macrophage infiltration with a focus on micro-anatomical localisation, reported that tumour islet or tumour-front infiltrating macrophages were associated with a good prognosis (Ohno *et al*, 2003; Forssell *et al*, 2007).

Because of the contradictory findings especially with NSCLC, our goal was to clarify the function of macrophages with regard to their micro-anatomical localisation in patients with NSCLC. Besides, to date, it is unclear which factors are important for macrophage infiltration. The epidermal growth factor receptor (EGFR) plays a key role in tumour growth, angiogenesis, and metastasis in NSCLC. In a study on breast cancer specimens, it was reported that macrophage infiltration was associated with EGFR protein expression (Leek *et al*, 2000). In addition, there was experimental evidence that both macrophages and tumour cells were necessary and sufficient for comigration and invasion by a colony-stimulation factor-1/EGF paracine loop (Goswami *et al*, 2005). Based on these findings, we investigated whether the macrophage infiltration profile, in NSCLC, was associated with EGFR mutations, gene copy number or protein expression.

The purpose of this study was to investigate the prognostic value of tumour-associated macrophages with a focus on the micro-anatomical localisation and to determine whether molecular changes in EGFR are associated with macrophage infiltration in patients with resected NSCLC.

## MATERIALS AND METHODS

### Clinical samples

Consecutive patients who underwent surgery including lobectomy, bilobectomy or pneumonectomy for NSCLC between January 1997 and December 1998 at Seoul National University Hospital, Seoul, Korea and with adequate samples for analyses were included in this study. Patients with squamous cell carcinoma, adenocarcinoma, adenosquamous cell carcinoma, and large cell carcinoma were eligible, but small cell lung cancer, sarcomas, and carcinoid tumours were excluded. A thorough medical record review for data including clinical and pathological characteristics such as age, gender, smoking history, histological subtypes, pathological stage, recurrence of NSCLC, and survival information was carried out. Patients who had smoked more than 100 cigarettes during their lives were defined as smokers.

This study was conducted in compliance with a study protocol that was approved by the Institutional Review Board of Seoul National University Hospital.

### Immunohistochemisty for CD68+ macrophages

The specimens for immunohistochemical analysis were formalin-fixed and paraffin-embedded. We used blocks containing only the advancing edge of the primary tumour. One 4 *μ*m section from each patient was cut onto glass slides. Briefly, the slides were dewaxed, rehydrated, washed, and subjected to microwave retrieval in a Tris/EDTA buffer (pH 9.0); they were then immersed in 3% H_2_O_2_. Anti-CD68 antibody (M0876, Dako, Denmark; 1 : 100) was used and immunoexpression was detected using a peroxidase-labelled streptavidin–biotin complex according to the manufacturer's instructions. Meyer's hematoxylin counterstaining was performed on the histology slides.

### Evaluation of macrophage infiltration

The number of CD68+ macrophages was analysed as previously described (Welsh *et al*, 2005). Briefly, the five most representative high-power fields (× 400) per slide were selected using an Olympus BX51TF microscope (Olympus, Japan). The area of the tumour islet and surrounding stroma were measured using LEICA IM50 image analysis software (Version 4.0, LEICA Microsystems Imaging Solutions, Cambridge, UK). The number of CD68+ nucleated cells was counted manually and expressed as cells mm^3^. For further analysis, the data were divided into two groups with values above the median or below. The median was chosen as a cutoff value because the median was the best fitting cutoff when we compared with different cutoffs including the mean or the presence/abscence of countable macrophages. To evaluate the validity of the analysis, the area measurement and counting were repeated 3 weeks later with 20 cases. All analyses were performed blind with respect to the clinical outcomes.

### DNA sequencing for detection of EGFR mutations

Mutation analysis of EGFR exons 18, 19 and 21 was performed as previously described (Han *et al*, 2005). The frequency of EGFR mutations and their correlation with survival in this study subjects was previously described in part (Lee *et al*, 2007). Briefly, DNA was extracted from five 5-*μ*m paraffin sections, containing a representative portion of each tumour block, using QIAamp DNA Mini kits (Qiagen, Hilden, Germany). One hundred nanograms (ng) of DNA were amplified in a 20 *μ*l reaction solution containing 2 *μ*l of 10 × buffer (Roche, Mannheim, Germany), 1.5 mM of MgCl_2_, 0.3 *μ*M of each complementary primer, 250 *μ*M of deoxynucleoside triphosphate, and 2.5 units of DNA polymerase (Roche). Amplifications were performed using a 5 min initial denaturation at 94°C; followed by 30 cycles of 1 min at 94°C, 1 min at 55°C, and 1 min at 72°C; and a 10 min final extension at 72°C. Polymerase chain reaction products were purified using a QIAgen gel extraction kit (Qiagen) and DNA sequenced using an ABI-PRISM BigDye Terminator v3.1 (Applied Biosystems, Foster, CA, USA) with both forward and reverse sequence-specific primers. Sequence data were generated using an ABI PRISM 3100 DNA Analyser (Applied Biosystems), and sequences were analysed using Sequencer 3.1.1 software (Applied Biosystems) to compare variations.

### Tissue microarray construction

For evaluation of the EGFR gene copy number and protein expression, we constructed a tissue microarray. Slides of tumour samples stained with hematoxylin and eosin were reviewed by two pathologists independently (HSM and DHC) and representative areas were marked. Core tissue biopsy specimens (2 mm in diameter) in duplicate were obtained from individual paraffin-embedded samples (donor blocks) and arranged in a new recipient paraffin block (tissue array block) using a trephine apparatus (SuperBioChips, Seoul, Korea). Serial sections from the tissue microarray block were analysed for EGFR gene copy number and protein expression.

### Fluorescence *in situ* hybridisation

The EGFR gene copy number was analysed using fluorescence *in situ* hybridisation (FISH). A dual-probe hybridisation was performed on 3-*μ*m-thick sections from tissue microarray blocks using the LSI EGFR SpectrumOrange/CEP7 SpectrumGreen probe set (Vysis, IL, USA) as described elsewhere (Cappuzzo *et al*, 2005). Fluorescence *in situ* hybridisation signals for each locus-specific FISH probe were assessed using an Olympus BX51TRF microscope (Olympus, Japan) equipped with a triple-pass filter (DAPI/Green/Orange; Vysis, IL, USA). The entire area of the tissue microarray core was evaluated in each case, and as many nonoverlapping nuclei as possible were assessed for an orange colour (marker) and green (reference) signals by a single pathologist (HSM) blinded to any information about the patients. The EGFR gene copy number was classified into six categories as described previously (Cappuzzo *et al*, 2005): (1) disomy (⩽2 copies in >90% of cells); (2) low trisomy (⩽2 copies in ⩾40% of cells, three copies in 10–40% of the cells, ⩾4 copies in <10% of cells); (3) high trisomy (⩽2 copies in ⩾40% of cells, three copies in ⩾40% of cells, ⩾4 copies in <10% of cells); (4) low polysomy (⩾4 copies in 10–40% of cells); (5) high polysomy (⩾4 copies in ⩾40% of cells) and (6) gene amplification (defined by presence of tight EGFR gene clusters and a ratio of EGFR gene to chromosome of ⩾2 or ⩾15 copies of EGFR per cell in ⩾10% of analysed cells). Based on the EGFR gene copy number, patients were further classified into two groups, EGFR–FISH positive (high polysomy and gene amplification) or EGFR–FISH negative (disomy, low trisomy, high trisomy, and low polysomy).

### Immunohistochemistry for EGFR protein expression

Epidermal growth factor receptor protein expression was evaluated by immunohistochemistry (IHC) on 4-*μ*m-thick sections from tissue microarray blocks using mouse anti-human EGFR, clone 31G7 monoclonal antibody (Zymed laboratories, CA, USA) was used with the labelled streptavidin-biotin complex staining method (LSAB kit, DAKO, CA, USA). Antigen retrieval was achieved by proteinase K digestion for 10 min, and the primary antibody was applied at a dilution of 1 : 50. Two trained pathologists (HSM and DHC), without knowledge of patient clinical data, scored the tumour staining. Each cell was scored as 0, 1, 2, or 3, which corresponded to negative, weak, moderate and strong staining intensities. The percent stained cells were determined and a final histochemical score (H-score) was calculated by summing the products of the staining intensities (0–3) and their distributions (0–100%). The H-scores ranged from 0 to 300. For statistical analysis, patients were classified into two groups, EGFR-IHC positive (H-score; 151–300) or EGFR-IHC negative (H-score: 0–150).

### Statistical analysis

The statistical analyses of categorical variables were done using the Pearson's *χ*^2^ test or Fisher's exact test where appropriate. The Spearman's rank correlation method was used to test for correlations between paired variables. The Kaplan–Meier survival analysis was used to estimate the overall survival (Kaplan and Meier, 1958), and comparisons between groups were done with the log-rank test. Multivariate logistic analyses were carried out using a stepwise Cox regression model for overall survival to identify independent variables and to adjust for baseline characteristics. Two-sided *P*<0.05 was considered significant. All analyses were performed using SPSS Version 12.0 for Windows software package (SPSS Inc., IL, USA).

## RESULTS

### Patient characteristics

There were 144 patients included in this study; their clinical and pathological characteristics are summarised in Table 1. The mean patient age was 60.4 years (s.d., 8.9) and 74 percent of patients were male. Fifty-five percent of patients had stage I disease, 17 percent stage II, 29 percent stage IIIA, and 9 percent stage IIIB or IV. Squamous cell carcinoma histology was present in 51 percent of patients. Complete resection was performed in 94 percent of patients. Twenty percent of patients received adjuvant treatments including radiation (17%) and chemotherapy (3%). The overall 5-year survival rate was 51%.

### CD68+ macrophage expression

**Figure 1:**
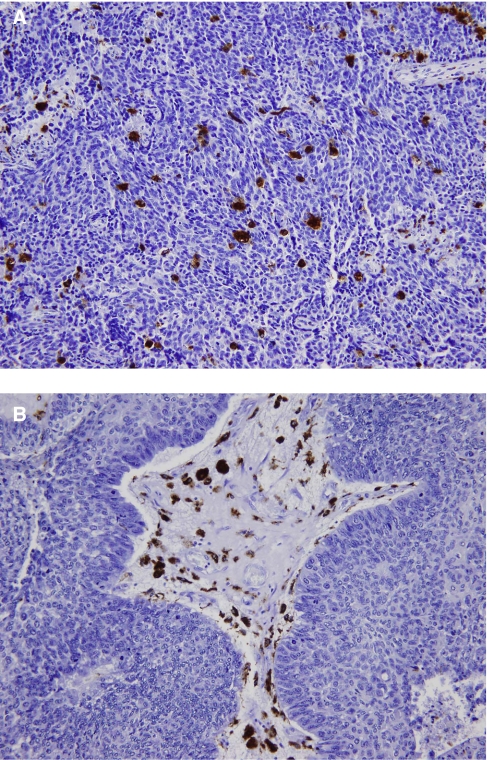
Immunohistochemistry demonstrating the presence of CD68+ macrophages (brown) in (**A**) tumour islet and (**B**) tumour stroma (× 200).

IHC demonstrated CD68+ macrophages clearly as dark brown-stained nucleated cells in both the tumour islet and stroma (Figure 1). The median number of macrophages in the tumour islet was 28 cells mm^3^ (range, 0–1823) and in the stroma, it was 223 cells mm^3^ (range, 0–1218). When we repeated the macrophage count in 20 cases to test for validity of the initial counts, good correlations were found for both the tumour islet (correlation coefficient 0.975, *P*<0.0001) and the stroma (correlation coefficient 0.962, *P*<0.0001).

### EGFR mutation, gene copy number, and protein expression

Epidermal growth factor receptor mutations were found in 29 patients (20.1%). including one patient with exon 18 (E709V) mutations, 23 with exon 19 (deletion) mutations, and six with exon 21 (L858R) mutations. One patient had exon 18 and 21 mutations simultaneously. Twenty-six (44%) of the adenocarcinoma patients had EGFR mutations, whereas only three (3.5%) nonadenocarcinoma patients had EGFR mutations. Only one patient received palliative gefitinib treatment after disease recurrence and had a partial response: this patient had a below median number of tumour islet macrophages.

Twenty-seven (19%) patients had EGFR–FISH positive tumours and 31 (22%) had EGFR-IHC positive tumours. The frequency of EGFR–FISH positive cases was higher in EGFR mutation positive (*P*=0.008) or EGFR-IHC positive tumours (*P*<0.00001). However, a correlation between the EGFR mutation frequency and the EGFR-IHC status was not significant (*P*=0.110).

### Correlation of macrophage infiltration with clinical and pathological variables and EGFR status

An analysis of a correlation between the tumour islet or stromal macrophage counts and other clinical and pathological variables revealed no significant associations. The EGFR mutations, gene copy number, and protein expression were not associated with the number of macrophages (Table 2).

### Survival analysis according to the macrophage counts

**Figure 2:**
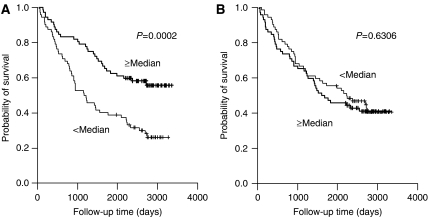
Kaplan–Meier overall survival curves stratified according to the tumour islet macrophage density (**A**) and stromal macrophage density (**B**).

**Figure 3:**
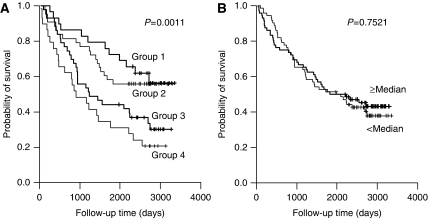
Kaplan–Meier overall survival curves stratified according to the tumour islet and stromal macrophage density (**A**) and total (tumour islet plus stromal) macrophage density (**B**). Group 1 (*n*=29): patients with high tumour islet and low stromal macrophage density; Group 2 (*n*=43): patients with high tumour islet and high stromal macrophage density; Group 3 (*n*=43): patients with low tumour islet and low stromal macrophage density; Group 4 (*n*=29): patients with low tumour islet and high stromal macrophage density.

Patients with high tumour islet macrophage density survived longer compared to the patients with low tumour islet macrophage density group (5-year overall survival rate was 63.9%. for high tumour islet macrophage *vs* 38.9% for low tumour islet macrophage, *P*=0.0002, Figure 2A). The stromal macrophage counts were not found to be significantly associated with survival (*P*=0.6306, Figure 2B). However, when we analysed survival according to both the tumour islet and the stromal macrophage count, high stromal macrophage counts were associated with poor survival within high or low tumour islet macrophage group (*P*=0.0011, Figure 3A). The total (tumour islet plus stromal) macrophage count was not significantly correlated with survival (*P*=0.7521, Figure 3B). When we divided the patient groups into adenocarcinoma and non-adenocarcinoma cases, the tumour islet macrophage count was a significant prognostic factor for both patient groups (*P*=0.0217, 0.0033, respectively).

### Survival analysis according to the clinical and pathological variables

**Figure 4:**
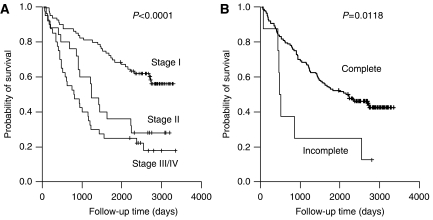
Kaplan–Meier overall survival curves stratified according to the TNM stage groups (**A**) and complete resection status (**B**).

Among the clinical and pathological variables, the TNM stage, and complete resection status were significant prognostic factors (*P*<0.0001 and *P*=0.0118, respectively, Figure 4A and B). However, the histology group (adenocarcinoma *vs* nonadenocarcinoma), gender (male *vs* female), age (<60 years *vs* ⩾61 years), and smoking status (smoker *vs* nonsmoker) were not significantly associated with survival. In addition, EGFR mutations, gene copy number, and protein expression showed no significant correlation with survival.

### Multivariate analysis for independent prognostic factor

The multivariate Cox proportional hazard analysis, with the variables significant on the univariate analysis (tumour islet macrophage counts, TNM stage group, and complete resection status), revealed that the tumour islet macrophage count was an independent favourable prognostic factor (hazard ratio 0.471, 95% confidence interval 0.300–0.740, *P*=0.001). In addition, the TNM stage was also an independent prognostic factor (*P*<0.0001); however, the complete resection status was not (Table 3). The multivariate analysis that incorporated additional covariates including age, gender, smoking status, and stromal macrophage count also showed that the tumour islet macrophage count was an independent favourable prognostic factor (hazard ratio 0.401, *P*<0.0001).

## DISCUSSION

The result of this study showed that a high number of tumour islet macrophages were a favourable independent prognostic factor for survival in patients with resected NSCLC. These findings are consistent with other studies on the microlocalisation of macrophages in NSCLC, gastric cancer, and colon cancer (Ohno *et al*, 2003; Welsh *et al*, 2005; Forssell *et al*, 2007). All of these prior studies showed that macrophage infiltration in the tumour islet or tumour-front associated with a good prognosis. Considering the ‘macrophage balance theory’ (Mantovani *et al*, 1992) and preclinical evidence for a dual function of macrophages (Bonta and Ben-Efraim, 1993; Cui *et al*, 1994; Xiong *et al*, 1998; Leek and Harris, 2002), there may be at least two kinds of macrophages in tumour tissue. One type may have a tumour cell killing function by production of cytotoxic molecules such as nitrogen oxide and tumour necrosis factor-*α*, and the other may have a tumour cell enhancing function by the secretion of growth factors and/or angiogenic molecules. In this context, tumour islet macrophages may represent the tumour killing macrophages. However, the underlying mechanisms of the different macrophage functions, according to the micro-anatomical localisation, remain to be elucidated. Recently, an *in vitro* coculture study suggested that tumour cell inhibition by macrophages was partially dependent on cell-to-cell contact (Forssell *et al*, 2007). To improve our knowledge of immunohistochemical findings on clinical samples, future functional studies with tumour-associated macrophages are needed.

In an effort to discern the molecular factors that influence macrophage distribution, we tested the relationship between the EGFR mutations, gene copy number, and protein expression with macrophage counts. However, we could not demonstrate any correlation between the EGFR variables studied and macrophage distribution. Preclinical evidence has suggested that EGFR mutations might be related to macrophage migration (Goswami *et al*, 2005). However, based on our study results, EGFR mutations, gene copy number, and protein expression does not appear to be associated with the distribution of tumour-associated macrophages.

The result of this study suggested that the tumour islet macrophage count is an important clinical determinant of patient prognosis after surgical resection of NSCLC, independent of the TNM stage. Therefore, decisions with regard to adjuvant chemotherapy, especially in the early stage of disease, should include tumour islet macrophage counts. However, for this approach to be used in the clinical setting, the standardisation of a counting method and a clinically practical cutoff value other than a median number should be established.

In conclusion, tumour islet macrophage infiltration was identified as a strong favourable independent prognostic marker for survival in patients with resected NSCLC. However, EGFR mutations, gene copy number, and protein expression did not correlate with macrophage infiltration in our patient group. Future functional studies with tumour-associated macrophages and clinical studies evaluating tumour islet macrophage counts are warranted.

## Figures and Tables

**Table 1:** Baseline patient characteristics

**Characteristics**	**No of patients (N=144)**	**%**
*Sex*		
Male	106	73.6
Female	38	26.4
		
*Age, years*		
Mean	60.4	
s.d.	8.9	
		
*TNM stage*		
IA	25	17.4
IB	54	37.5
IIA	8	5.6
IIB	17	11.8
IIIA	27	18.8
IIIB	11	7.6
IV	2	1.4
		
*Histology*		
Adenocarcinoma (Total/BAC)	59/15	41.0/10.4
Squamous cell carcinoma	73	50.7
Adenosquamous	8	5.6
Large cell carcinoma	4	2.8
		
*Smoking history*		
Never smoked	46	31.9
Smoked	98	68.1
		
*Resection*		
Complete	136	94.4
Incomplete	8	5.6
		
*Adjuvant treatment*		
None	115	79.9
Radiation	24	16.7
Chemotherapy	5	3.4

Abbreviations: BAC=bronchioloalveolar carcinoma; s.d.=standard deviation.

**Table 2:** Association of macrophage counts with clinicopathologic and EGFR status

**Characteristics**	**Tumour islet macrophage**			**Stromal macrophage**		
	**<Median**	**⩾Median**	***P***	**<Median**	**⩾Median**	***P***
Total patients (*N*=144)	72	72		72	72	
*Sex*						
Male	51	55	0.285	48	58	0.088
Female	21	17		24	14	
						
*Age*						
<60	31	38	0.158	39	30	0.182
>60	41	34		33	42	
						
*Histologic type*						
Adenocarcinoma	34	25	0.175	32	27	0.498
Nonadenocarcinoma	38	47		40	45	
						
*TNM stage*						
Stage I	35	44	0.264	37	42	0.685
Stage II	13	12		13	12	
Stage III/IV	24	16		22	18	
						
*Resection*						
Complete	65	71	0.063	69	67	0.719
Incomplete	7	1		3	5	
						
*Smoking status*						
Nonsmoker	22	24	0.858	25	21	0.592
Smoker	50	48		47	51	
						
*EGFR mutation*						
Absent	56	59	0.678	57	58	1.000
Present	16	13		15	14	
						
*EGFR FISH*						
Negative	50	57	0.410	51	56	0.617
Positive	16	11		15	12	
No data	6	4		6	4	
						
*EGFR IHC*						
Negative	55	55	0.833	57	53	0.681
Positive	15	16		14	17	
No data	2	1		1	2	

Abbreviations: EGFR=epidermal growth factor receptor; FISH=fluorescence *in situ* hybridization; IHC=immunohistochemistry.

**Table 3:** Results of Cox regression analysis predicting 5-year survival

**Factor**	**Hazard ratio**	**95% confidence interval**	***P***
Tumour islet macrophage density	0.471	0.300–0.740	0.001
			
*TNM stage*			
I	0.304	0.180–0.511	<0.0001
II	0.701	0.389–1.262	0.236
III and IV	1.00		
			
Complete resection status	1.164	0.514–2.638	0.716
